# Sorting of Single Biomolecules based on Fourier Polar Representation of Surface Enhanced Raman Spectra

**DOI:** 10.1038/srep20383

**Published:** 2016-02-02

**Authors:** Aymeric Leray, Thibault Brulé, Mickael Buret, Gérard Colas des Francs, Alexandre Bouhelier, Alain Dereux, Eric Finot

**Affiliations:** 1 Laboratoire Interdisciplinaire Carnot de Bourgogne, UMR 6303 CNRS, Université Bourgogne Franche-Comté, 21000 Dijon (France)

## Abstract

Surface enhanced Raman scattering (SERS) spectroscopy becomes increasingly used in biosensors for its capacity to detect and identify single molecules. In practice, a large number of SERS spectra are acquired and reliable ranking methods are thus essential for analysing all these data. Supervised classification strategies, which are the most effective methods, are usually applied but they require pre-determined models or classes. In this work, we propose to sort SERS spectra in unknown groups with an alternative strategy called Fourier polar representation. This non-fitting method based on simple Fourier sine and cosine transforms produces a fast and graphical representation for sorting SERS spectra with quantitative information. The reliability of this method was first investigated theoretically and numerically. Then, its performances were tested on two concrete biological examples: first with single amino-acid molecule (cysteine) and then with a mixture of three distinct odorous molecules. The benefits of this Fourier polar representation were highlighted and compared to the well-established statistical principal component analysis method.

Biosensors are analytical devices measuring the concentration of specific analytes. They are ubiquitous in many fields (e.g. for detecting pesticides^1^,^2^, for analysing food quality^3^,^4^ and environment^5^ or for biomedical diagnostics^6^,^7^,^8^). Optical biosensors receive increasing interest due to their high sensitivity and selectivity (for a review, see^9^). This unique selectivity is enabled by spectroscopically analysing inelastic optical responses such as fluorescent emission or Raman scattering. However, due to the extremely small scattering cross section, Raman spectroscopy was for a long time limited to steady state or slow dynamic process^10^ in bulk materials or highly concentrated solutions^11^,^12^. For the last twenty years, the interest in Raman spectroscopy has been renewed when surface enhanced Raman scattering (SERS) has been demonstrated as an effective label-free spectroscopy for identifying and classifying single molecules^13^,^14^,^15^,^16^,^17^,^18^. The SERS effect is characterized by a significant enhancement of the Raman signal emitted by molecules present in close proximity of metallic nanoparticles. When excited by an appropriate laser frequency, localized surface plasmon resonances sustained by the metallic nanoparticles provide a high enhancement of the electric field intensity that can reach several orders of magnitude (at least 10^6^).

In addition to their single molecule sensitivity, SERS sensors, by nature, provide a highly specific spectroscopic response also at the individual molecules level^13^,^19^. In practice, the combination of a high sensitivity with a high specificity result in the acquisition of a large number of distinct spectra. These numerous spectra may reflect the fact that either a large number of distinct molecules are present in the medium or that the analyte can be described by numerous states such as distinct conformations or positions in the SERS active region. In all cases, a reliable classification of these numerous SERS spectra is essential for accurately interpreting the output of the sensor.

The most sophisticated and effective methods for classifying SERS spectra are based on a predetermined library of spectra. They are called supervised methods and include linear discriminant analysis (LDA)^20^,^21^ and partial least squares discriminant analysis (PLS-DA)^22^,^23^. However, building a valid classification model requires the acquisition of a training set of samples. Furthermore, these classification models are only valid for retrieving a known molecule with a given SERS based sensor and might not be adaptable to other platforms.

An alternative sorting tool exists to group SERS spectra by similarities into unknown classes without the need of prerequisite models. This multivariate analysis called principal component analysis (PCA) is used to reduce the dimensionality of measured SERS spectra into few principal components. The advantage of this strategy is that no *a priori* knowledge is required for data analysis. PCA has thus been largely used to regroup similar SERS spectra into clusters^24^,^25^,^26^,^27^,^28^. However, this method is limited to disentangle relatively simple mixture because it is graphically bound to three principal components.

In this work, we propose an original method that overcomes the limitations of PCA. Our representation sorts molecular spectra in a fast and visual way and provides mixture proportion without the need of precalibration step or reference spectra. Briefly, our approach is to reduce SERS spectra into a Fourier polar representation. Each spectrum is then transformed to a pole whose coordinates (modulus *m* and phase *φ*) correspond to the first component of its Fourier transform. Therefore, all SERS spectra are visualized into a scatter diagram. The geometry of the pool of poles gives an indication on the classification (e.g. binary or ternary mixture).

This strategy was initially applied in fluorescence lifetime imaging microscopy^29^,^30^,^31^ and was recently used for unmixing fluorescence spectral images^32^ and for spatially segmenting hyperspectral stimulated Raman scattering images^33^. However, to the best of our knowledge, it has never been applied to SERS experiments. Due to the large enhancement factor allowing single molecule measurements, SERS spectra are usually composed of multiple peaks with highly temporally fluctuating intensity. In this work, we have considered these specific issues of SERS experiments by investigating for instance the influence of the enhancement factor on the Fourier polar representation of multiple peaks spectra.

The paper is organized as followed. We first describe the principle of the Fourier polar representation and discuss the meaning of poles for single and multiple peaks spectra. We then apply it to small biomolecules, which are more difficult to be detected. We first consider one amino acid, the cysteine, whose levels are raised in Alzheimer’s and Parkinson’s diseases^34^. Experimental SERS spectra of single cysteine molecule are analysed with Fourier polar representation. We also apply it to a ternary mixture of distinct small odorous molecules: acetoin (butter odour), eugenol (clove odour) and IBMP (green pepper odour) which have been largely used for studying biological mechanisms involved in the perception of smell or taste and flavour^35^. We finally discuss the advantages and limitations of this representation compared to the principal component analysis.

## Theoretical Framework

### Case of single peak SERS spectrum

A phasor (contraction of phase vector) is a complex number representation written as a complex exponential *m*e^*iφ*^ where *m* is the vector modulus and *φ* is the phase. Each SERS spectrum *I*(λ) is converted to a phasor by using a Fourier transform. The (u_1_;v_1_) coordinates corresponding to the real and imaginary parts of the first component of the Fourier transform are expressed as


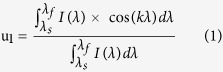



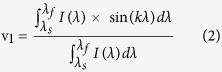


where *λ*_*s*_ and *λ*_*f*_ are respectively the start and final wavelengths of the spectral range and *k* is the angular wave number defined as *k* = *2π/(λ*_*f*_ *−* *λ*_*s*_).

The polar coordinates corresponding to modulus *m* and phase *φ* are obtained from the (u_1_;v_1_) coordinates with


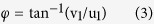



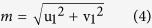


Take for instance a SERS spectrum composed of a single peak at wavelength *λ*_*0*_ approximated by a Dirac delta function *I*(*λ*) = *I*_*0*_*×*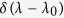. The coordinates are simply given by


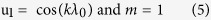



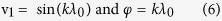


**Figure 1:**
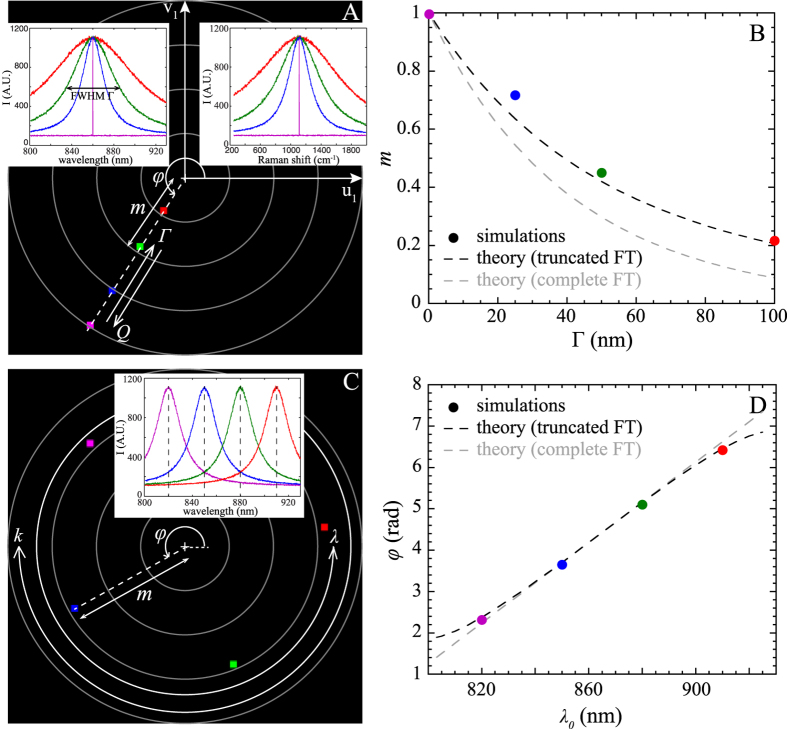
(**A**) Fourier polar representation of 4 single Raman band centred at 860 nm with increasing the full-width at half-maximum Γ of the Raman bands. (**B**) Corresponding modulus *m* as a function of Γ. When increasing Γ, poles come closer to origin. Theoretical modulus *m* is plotted for an infinite (in grey dotted line) and finite wavelength range (in black dotted line). (**C**) Fourier polar representations for 4 single Raman band of the same Γ but shifted with different *λ*_*0*_. Poles turn anti-clockwise when increasing *λ* and clockwise when increasing *k*. Corresponding phase shifts for these peaks are represented in (**D**). Theoretical phase *φ* is plotted for an infinite (in grey dotted line) and finite wavelength range (in black dotted line).

The equality u_1_^*2*^ + v_1_^*2*^ = 1 corresponds to the equation of a circle of radius 1 centred at the origin [0, 0]. In other words, an ideal single band SERS spectrum is represented by a pole localized on a circle centred at the origin with a radius of 1 (cf. Fig. 1A). Only *φ* provides information about the resonant wavelength of the band.

If we consider more realistic SERS spectra where Raman bands are modelled with Lorentzian functions (with full-width at half-maximum FWHM of Γ) given by: 
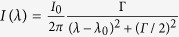
, the coordinates for an infinite spectral measurement width are given by


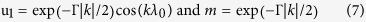



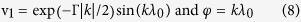


A Raman band (with a finite quality factor) is then represented by a pole located inside the unit circle and its modulus (*m *< 1) decreases exponentially when Γ increases (as indicated in Fig. 1A). Realistic Raman peaks with Γ = 1 nm leads to a modulus *m* > 0.97.

Equations 7 and 8 (which are represented in grey in Fig. 1B,C) are only fully valid for an infinite spectral measurement width. Small differences between the theoretical expressions and our Monte Carlo simulations (black dots) are explained by the finite wavelength range [*λ*_*s*_*;λ*_*f*_] used to simulate the SERS spectra. In this case, the analytical calculations of the polar coordinates are not obvious. Results shown by the black dotted lines in Fig. 1B,D are obtained from computational study for SERS spectra modelled by Lorentzian functions with a finite spectral measurement width. The numerical results, performed with Mathematica 10 (Wolfram Research, Inc), are now in excellent agreement with Monte Carlo simulations.

### Case of multiple peaks SERS spectrum

A full Raman spectrum composed of *N* peaks is ideally modelled by 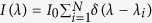. The [u_1_; v_1_] coordinates are


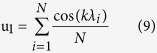



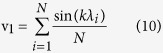


These coordinates correspond thus simply to the linear combination of the [u_1_;v_1_] values of all individual SERS peaks. Calculations of the modulus *m* and the phase *φ* are easily feasible analytically for two peaks of identical height with wavelengths *λ*_*1*_ and *λ*_*2*_ (*λ*_*2*_ > *λ*_*1*_)


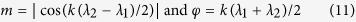


**Figure 2:**
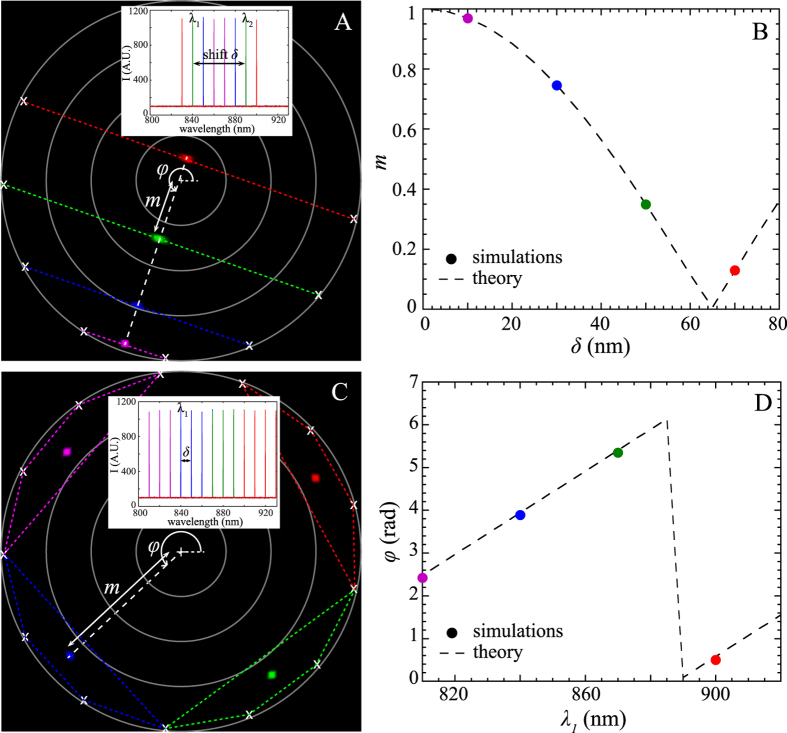
(**A**) Fourier polar representation for 4 double peaks spectra with variable spectral gap *δ*. (**B**) Simulated (coloured points) and theoretical (dotted lines) modulus *m* are plotted as a function of *δ.* (**C**) Fourier polar representation of four-peaks combs spectra separated with the same *δ* but shifted in wavelength. Corresponding *φ* as a function of the starting wavelength of the peak comb are compared in (**D**) with theoretical expectations.

In this case, the Fourier polar representation is localized on the middle of the segment formed by the coordinates of each individual peaks (Fig. 2A). These theoretical expressions are in excellent agreement with simulations (cf. Fig. 2B). When *N* ≥ 3, an analytical calculation of *m* and *φ* is not trivial and numerical computation is required.

However, some simple cases can still be detemined analytically. For instance, if we consider a comb of *N* Raman peaks of identical height that are uniformly separated by a fixed spectral shift *δ* and with first wavelength *λ*_*1*_, the modulus and the phase shift are given by


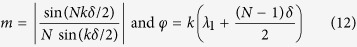


*φ* is governed by the central wavelength of the comb whereas the modulus is related to the number of peaks in the comb (at fixed *δ*, *m* decreases when *N* increases).

For *N* = 4, the polar representation is localized inside the unit circle (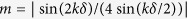 < 1) and more precisely inside the polygon formed by [u_1_;v_1_] values of ideal individual single peak SERS spectra (Fig. 2C). The phase shift 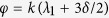 matches satisfactorily the simulated results (Fig. 2D).

### Case of a mixture

A SERS spectrum does not necessarily stem from only one single molecule or even one molecular state. For instance, several molecular states of the same molecule may be activated simultaneously in the hotspot. The corresponding acquired spectrum will be a combinatory assembly of the SERS fingerprints of each molecular state.

To illustrate this situation, we first consider a binary mixture of two molecular states with proportions *p*_*1*_ and *p*_*2*_. If only one molecule is present in the enhancement site, the SERS measurements will only give two distinct spectra (*p*_*1*_ = 1 or *p*_*2*_ = 1) and the corresponding polar representation is a segment with two poles (Table 1). When two (and respectively three) states are mixed in SERS measurements, the corresponding polar representation is now a segment with 3 (and respectively 4) poles (whose proportions are listed in Table 1).

A ternary mixture of three states leads to a more complex combinatory analysis (Table 1). The 3 principal poles, corresponding to the isolated pure state, form a triangle. The number of poles can be 3, 6 and 10 according to the number of possible molecules (1, 2 and 3) detectable in the SERS active volume at the same time. The pool of poles in a ternary mixture has the shape of a triangle. The advantage of the Fourier polar representation becomes obvious. The polar plot graphically identifies the number of components of the mixture as well as their proportions.

In the next section, these theoretical considerations will be validated by simulations and experiments using both a binary and a ternary mixture.

## Results

### Binary mixture: Monte Carlo simulations

**Figure 3:**
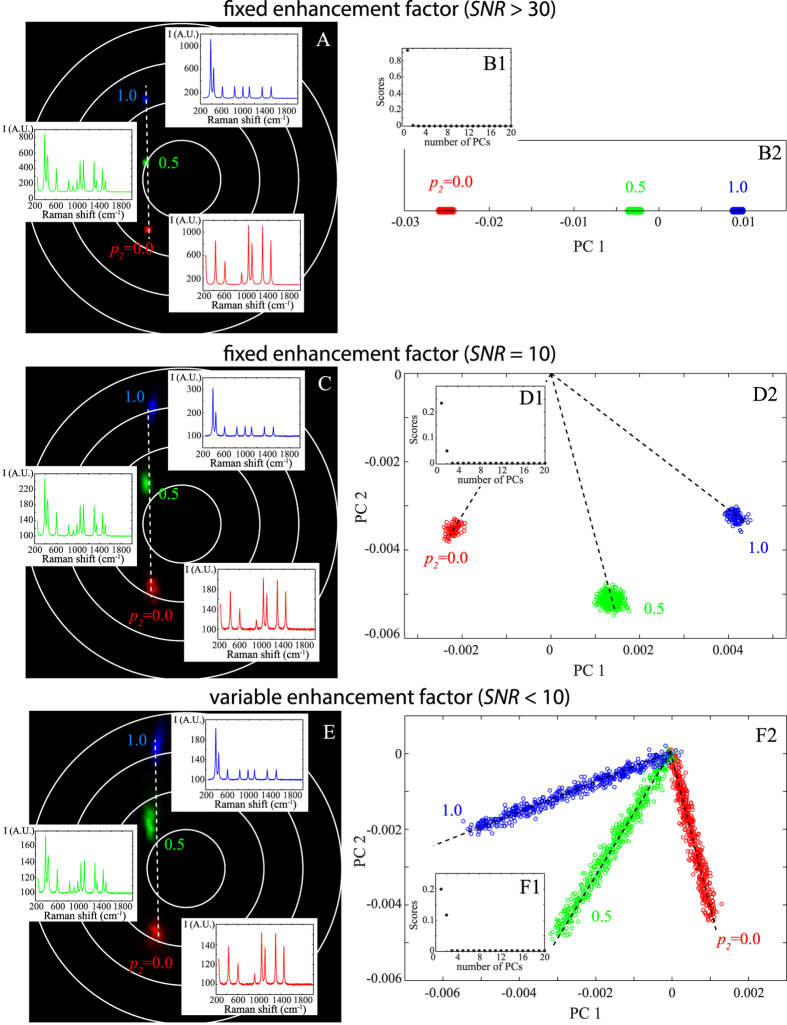
Comparison between the Fourier polar representation (left) and the PCA (right) applied to simulated spectra constituted of a binary mixture with varying mix ratio (*p*_*2*_ = 0; 1/2 and 1). The influence of the signal to noise ratio (*SNR*) (at the top) and the enhancement factor *G* (at the bottom) are illustrated. At high *SNR* and fixed *G*, the corresponding polar representation and PCA are indicated respectively in (**A,B2**). For low SNR and fixed *G*, polar representation and PCA are indicated in (**C,D2**). When the enhancement factor is computationally modified, the Fourier polar representation (**E**) is almost not affected whereas PCA (**F2**) exhibits branches. The scores of PCs are indicated for each case in B1, D1 and F1.

For a deeper analysis of a binary mixture, we performed Monte Carlo simulations (see Material and Methods). We consider two molecular states with characteristic Raman spectra reported in blue and red in Fig. 3A. We first study high signal to noise ratio SERS spectra (*SNR* > 30). As previously mentioned, when two or three molecules are passing through the SERS active volume, the Fourier polar representation depicts a line with 3 or 4 poles (Fig. 3A). The poles at both ends of the segment correspond to the SERS spectra of individual molecular state (not mixed) and the internal poles (in green) result from the linear combination of each principal state. Note that the length of the segments corresponds directly to the mix ratio.

The Fourier polar representation was also compared with the well-established PCA method. As a reminder, PCA reduces the dimensionality of the measured SERS spectra by converting a set of correlated data into linearly orthogonal variables called principal components (PCs). PCA applied to the previous Monte Carlo simulations shows that one PC is sufficient to describe the SERS spectra, and three color-coded families (blue, red and green) are successfully retrieved. However, there is no longer a direct relation between the proportion and the position of the poles.

Let us then consider more realistic SERS spectra with lower signal to noise ratio (*SNR* = 10). The Fourier polar representation still exhibits 3 aligned poles (Fig. 3C) but these poles are widened with an oblong shape. The green pole is no longer exactly positioned in the midpoint.

With the PCA, two PCs are now necessary for interpreting the SERS spectra that are still classified into 3 families (see Fig. 3D1,D2). The blue and red families correspond to the pure spectra (*p*_*2*_ = 0 and *p*_*2*_ = 1). They form an angle of almost 90° indicating that they are independent. Again, there is no direct relation between the proportion and the position of the poles.

In practice, molecules are extremely mobile inside the enhanced site implying that the total intensity of the SERS spectra is temporally fluctuating^36^ and is extremely dependent on the position of the molecule in the SERS active volume^37^,^13^. In order to take these fluctuations into account, we add a variable enhancement factor in our Monte Carlo simulations. In presence of fluctuations, the polar representation remains unaffected while the results of the PCA are modified (Fig. 3EF2). The previous 3 poles are now declined into branches.

### Binary mixture: experimental application on cysteine

**Figure 4:**
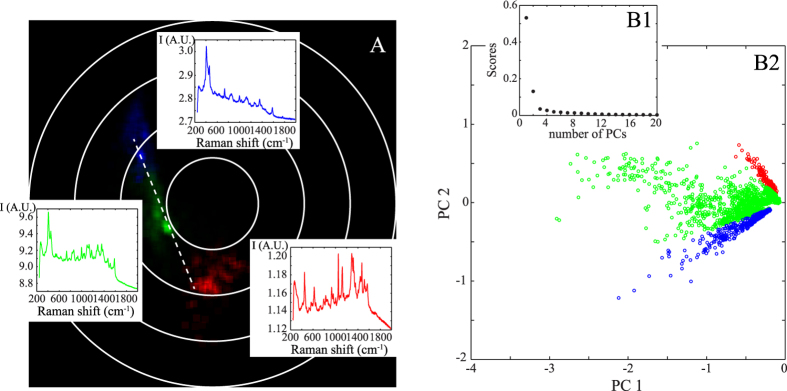
Comparison between Fourier polar representation (A) and PCA (B2) for analysing the experimental spectra of cysteine. Both PCA and polar representation are effective at sorting the spectra in three families coloured in blue, red and green. The corresponding Raman spectra are also indicated. As shown in (**B1**), two PCs are used for characterizing the experimental data.

To validate our previous results deduced from Monte Carlo simulations, we apply the same methodology to experimental SERS spectra acquired when cysteine is circulated in a microfluidic sensor. We demonstrated in a previous work that cysteine is likely to form a dimer (i.e. cystine) and thus can be treated as a binary mixture^13^. The corresponding polar representation reported in Fig. 4A emphasizes three main families colorized in blue, red and green corresponding to three distinct spectra. These families are almost localized on a line, and the extremities are formed from the blue and red families. This representation indicates that the red and blue families are nearly independent and that the green family is composed of a linear combination of the red and blue families (as suggested from Monte Carlo simulations).

The PCA applied to the experimental SERS data leads to the same conclusion. As indicated in Fig. 4B1, two principal components are necessary for describing the data. The results of PCA are then represented in two dimensions and three families are isolated (Fig. 4B2). We identify two nearly orthogonal distinct families colorized in red and blue. The third green family is obtained from a linear mixture of these two principal red and blue spectra. The blue and red families have already been attributed to the SERS spectra of the cysteine and of its dimer, respectively^13^.

To conclude this section, when PCA can be reduced to a limited set of PCs, as in the case of single cysteine molecule, Fourier polar representation and PCA lead to similar sorting accuracy. We show in the following section the advantages of the polar representation when complex systems are investigated.

### Ternary mixture: experimental application on three different odorous molecules

**Figure 5:**
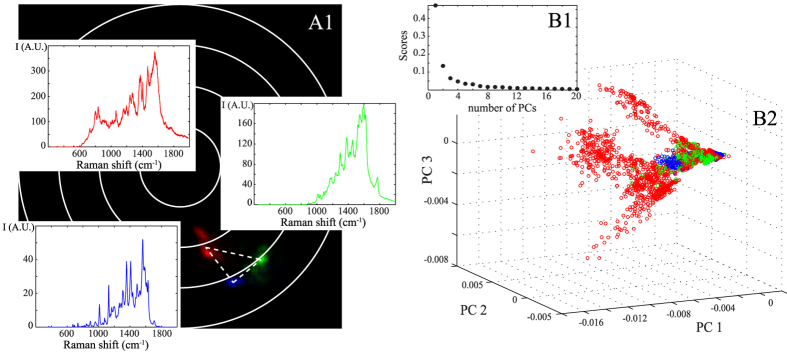
Comparison between the Fourier polar representation (A1) and the PCA (B2) applied on the experimental series of a mixture of three distinct aroma molecules: acetoin (indicated in blue), eugenol (in green) and IBMP (in red). The corresponding spectra are also shown in (**A1**). With PCA, 3 PCs are necessary for characterizing the experimental SERS spectra (see **B1**) but it is not possible to sort the three distinct molecules.

We finally experimentally study a ternary mixture composed of three distinct aroma molecules: IBMP (green pepper odour), acetoin (butter odour) and eugenol (clove door). We successively acquire 512 SERS spectra of each individual molecules circulating into a microfluidic SERS sensor. As indicated in Fig. 5B1, at least three PCs are necessary to perform principal component analysis. The corresponding three-dimensional PCA are represented in Fig. 5B2. Here, PCA is not able to distinguish the three odorous molecules. It seems that three principal components are not enough for sorting the ternary molecular mixture.

With the polar representation, it is not true anymore. As shown in Fig. 5A1, the Fourier polar representation forms a triangle with three poles, as expected theoretically for a ternary mixture with one molecule in the enhancement site (see Table 1). Because the polar coordinates are sensitive to both the number and the quality factor of the Raman bands, each type of molecule is well separated in the polar representation and can now be easily identified. The polar representation can thus be successfully used for sorting a ternary mixture of odorous molecules.

## Discussion

To determine the relevance of PCA and polar representation for sorting SERS spectra, we first clarify the advantages and drawbacks of each method.

PCA and polar representation are two powerful sorting methods, which do not require any *a priori* knowledge about the measured samples.

It is well known that the performance of any classification methods largely depends on the mathematical pre-treatment of spectra^20^. In this work, we have voluntarily restricted this pre-treatment to simple offset subtraction to be as close as possible to raw data. We could of course envision more complicated corrections for denoising and/or flattening SERS spectra that would surely modify our results. However, these corrections will equivalently modify the PCA and the polar representation and would not alter the comparison between these two methods.

We have shown that PCA is a robust approach for separating SERS spectra even if *SNR* is low. We previously applied it for discerning the different forms of cysteine^13^. However, the drawbacks of PCA include: (i) the lack of quantitative determination of the proportion in a binary mixture, (ii) the lack of reproducibility in the orientation of the branches preventing a comparison between distinct sets of experimental spectra and (iii) the failure to sort a ternary mixture. Finally, the PCA method is probably not well adapted for classifying SERS spectra of complex samples. When the number of principal components exceeds 3, the graphical representation of the PCA outcomes is difficult.

These drawbacks are largely overcome with the suggested polar representation. The phasor correctly classifies binary and ternary mixture even at low *SNR*. The polar representation provides quantitative information on the unknown mixture ratio. It is a 2D histogram displaying the real and the imaginary parts of the first component of the Fourier transform and it is thus applicable to any molecular SERS signatures (i.e. large and small proteins). Additionally, we have demonstrated that the complex modulus and the phase shift (*m* and *φ*) are relatively constant regardless of *SNR* for a given spectrum. The polar representation can thus be used to compare distinct sets of experimental data. We have shown that the phasor depends on both the number and the quality factor of peaks in the SERS spectra. Another advantage of the phasor is that the representation can be directly implemented on a standard computer enabling thus a classification of SERS spectra with an automatic identification of identical spectra during an experimental acquisition. We emphasize that this automation would not have been possible with other analysis methods including unsupervised grouping techniques such as clustering^38^.

Nevertheless, the Fourier polar representation is not free from weaknesses. Its major drawback is its extreme dependence on the offset subtraction that affects the first component of the Fourier sine and cosine transforms. It is therefore important to suppress fundamental frequency corresponding to the DC component (or offset). Otherwise, the calculated polar coordinates will be dependent on the level of this offset and the comparison between different sets of data will be impossible.

Here, we only calculated the polar coordinates of SERS spectra using the first component of the Fourier transform. In practice, our experimental SERS spectra are rich, and could thus be defined by a larger number of wavenumbers. We could then calculate the polar coordinates of SERS spectra with more components and plot several polar representations for each wavenumber^39^. It is worth noting that each polar representation will be different; in other words, the localization of the poles will be modified. The results indicated in Figs 1 and 2 would not be valid anymore as they are specific to the first component. An optimal wavenumber probably exists for maximizing the separation distance between spots. However, the precision of the polar representation is dependent on the amplitude of the Fourier components. This study was restricted to the first component because its amplitude is maximal, implying that the standard deviation is minimal. Furthermore, we experimentally showed that using the first component of Fourier transform was sufficient to correctly sort our data.

## Conclusions

In this work, we have introduced an alternative method for accurately and graphically sorting temporally fluctuating single molecule SERS spectra. This non-fitting method called Fourier polar representation does not require any prerequisite models or classes. We have explained its general principle and described theoretically the position of the poles according to both the number and the quality factors of the Raman bands. Graphical patterns obtained with binary and ternary molecular mixtures have been discussed. We have then demonstrated that the Fourier polar representation was equivalent to the well-established PCA method for sorting binary mixture. In addition, our method provides the proportions of the different components in the mixture, which is not possible with PCA. We have finally experimentally demonstrated the added value of our method on a binary mixture and a ternary mixture of different biomolecules. We stress that this Fourier polar representation was here applied for the first time to sort single molecule SERS spectra. In particular, using simple base-line correction of spectra, the Fourier polar representation was able to discriminate a ternary mixture of distinct odorous molecules, whereas this sorting was not obvious with PCA. Because of its ease of implementation, this non-fitting analysis method should be of global concern to the spectroscopic sensor community.

## Methods

### Monte Carlo simulations

SERS spectra with controlled parameters are simulated on a standard computer using a Monte Carlo approach. In brief, the number of Raman bands and their corresponding wavelengths are fixed. Then we used a random number generator to set the spectral intensity. The density probability function of the generator is a Poisson distribution with a mean corresponding to spectral intensity. In order to mimic as closely as possible to standard experimental conditions, we consider a spectral band of 130 nm starting at 800.65 nm and divided into 1024 pixels leading to a spectral resolution of 0.127 nm. The total intensity of SERS spectra *I*_*0*_ is Poisson distributed and thus signal to noise ratio (*SNR*) is shot-noise limited. We also added an offset (which is also Poisson distributed) and we have applied a variable enhancement factor (uniformly distributed between 0 and *G*) to each spectrum to take into account the mobility of the molecules into the active SERS area.

### Acquisition of SERS spectra

In order to measure exploitable SERS spectra, we use a raspberry-like gold nanoparticles template synthetized from a colloidal solution. The nanoparticles are deposited on a glass coverslip and assembled in a microfluidic cell (fabrication steps are described in^13^). Cysteine was diluted into PBS to obtain a concentration of 50 μg/mL. The aroma molecules: 3-isobutyl-2-methoxypyrazine (IBMP), 3-hydroxybutanone (acetoin) and eugenol were diluted into deionized water to a concentration of 10 μM. These different solutions are circulated into a microfluidic channel using a peristaltic pump generating a flow rate of 2 μL/min.

The microfluidic device is placed on a custom-built inverted confocal Raman microscope (scanned with a piezoelectric translation stage) coupled with a 785 nm laser diode. Back scattered spectra are acquired with a 60× water immersion objective (NA = 1.2, Nikon) at a frequency of 1 Hz by using a spectrometer composed of a 600 lines per millimeter grating associated with a cooled CCD camera (1024 × 1024 pixels).

### Data Analysis

SERS spectra are analysed by using two distinct methods: the PCA and the polar representation.

PCA is a well-established multivariate analysis technique converting the high dimensionality of the experimental data into a new variance weighted coordinate system with lower dimensions. The orthogonal linear transformation consists in determining the eigenvectors of the covariance matrix; the so-called principal components (PCs). In practice, each spectrum is first centred on its mean, and then the covariance matrix of the complete data set is calculated. The PCs and scores are resolved by using a homemade program designed with MATLAB.

The Fourier polar representation is programmed using MATLAB software. Fourier sine and cosine transforms are calculated for each spectrum and the intensity-weighted polar representation is displayed. Prior to each analysis (PCA and polar representation), SERS spectra are background corrected by estimating an average background from a part of the spectrum where the Raman signal is low and subtracting it from the full spectrum.

## Additional Information

**How to cite this article**: Leray, A. *et al.* Sorting of Single Biomolecules based on Fourier Polar Representation of Surface Enhanced Raman Spectra. *Sci. Rep.* **6**, 20383; doi: 10.1038/srep20383 (2016).

## Figures and Tables

**Table 1:**
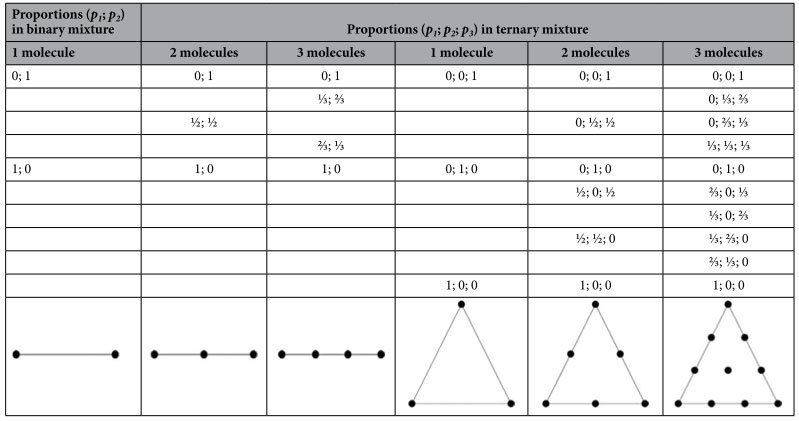
List of poles in a binary and ternary mixture of molecular states. The proportions *p*_*1*_*, p*_*2*_ and *p*_*3*_ of each vibrational state (1, 2, 3) are reported according to the number of molecules simultaneously present in the SERS active volume that will participate to the Raman spectrum (by assuming similar SERS intensity spectrum for each state).
